# The forgotten foundations: in core mental health services, no one can hear you scream

**DOI:** 10.1192/bjb.2018.79

**Published:** 2018-12

**Authors:** Andrew Moore

**Affiliations:** North Devon Community Mental Health Services (Sector A Team), Devon Partnership NHS Trust, UK

**Keywords:** Core mental health services, Five Year Forward View for Mental Health, community mental health teams, crisis teams, mental health in-patient units

## Abstract

The Five Year Forward View for Mental Health (FYFVMH) was a welcome development in the emerging ‘Parity of Esteem’ agenda, but focused mainly on a select few specialist services; much more limited attention was given to ‘core’ general adult and older age mental health services, such as community mental health teams, crisis teams and in-patient units. This relative policy vacuum, when combined with prolonged financial pressures and limited informatics, has left core services vulnerable and struggling to meet growing demands, with little sense of hope, in contrast to some of the newer, ‘shiny’ specialist services growing around them. Policy makers need to recognise the growing crisis and take action, ensuring that any sequel to the FYFVMH redresses this imbalance by clearly prioritising core services as the vital foundations of the larger whole-system. The potential benefits are huge and wide-ranging, but the harms of a second missed opportunity are perhaps even greater.

**Declaration of interest:**

A.M. works in a National Health Service general adult community mental health team and is an elected member of the Royal College of Psychiatrists General Adult Faculty Executive Committee.

The Five Year Forward View for Mental Health (FYFVMH)^1^ is now halfway through its lifespan, with policy makers assessing its early effects and pondering the sequel. Perhaps the biggest effect was simply having a specific mental health version of a Five Year Forward View. The ‘Parity of Esteem’ (for Mental Health) agenda is still relatively young, with considerable gaps between rhetoric and action, but the existence of a mental health version helped keep early momentum going. This must now accelerate, but with new priorities much more explicitly focused on ‘core’ general adult and older age mental health services, especially community mental health teams (CMHTs), crisis teams and acute in-patient units. Historically, these central foundations of the wider system have been vulnerable to ongoing, quiet erosion by chronic underfunding and systemic structural disadvantages (e.g. block contracts). The FYFVMH, despite enabling progress in some areas of its specific focus, was a painfully missed opportunity to take a more whole-service approach; this must not be repeated. There were some core service aspirations in the FYFVMH, such as enhanced 24/7 crisis teams, elimination of acute out-of-area placements and development of community pathways, but these were weakened by a lack of clear targets and/or specific ring-fenced funding. Despite early winners in the first FYFVMH, there are still some long-neglected losers. Future service planning urgently needs to redress this imbalance; systems are only as strong as their weakest parts, especially if they are core foundational components.

Quantitative analysis is dogged by informatics challenges. Despite a plethora of information sources, each has its limitations and none tell the whole story on their own. The National Health Service (NHS) England FYFVMH Dashboard^2^ is good in principle, but is inevitably focused on select areas of current policy, with minimal content on core services. Other sources include NHS Mental Health Benchmarking Data, ‘Fingertips’ Public Mental Health Data^3^ and a raft of *ad hoc* reports such as the Commission on Acute Adult Psychiatric Care,^4^ the Carter Report on Mental Health Services^5^ and Care Quality Commission reports.^6^ A 2017 Royal College of Psychiatrists survey of front-line clinicians, regarding perceived progress on the Acute Adult Psychiatric Care Commission recommendations, highlights deep and widespread concerns (personal communication). Combining all these, along with front-line experience, is not an easy task for dedicated researchers and policy leaders, and is almost impossible for busy clinicians. When attempted, an information bias emerges toward those areas that already have a national focus, making it all the harder for those services outside the current policy spotlight to make a coherent case for inclusion. Key information on core services can be hard to extract and harder still to interpret, giving a rather foggy, delayed view of a complex, changing landscape, leaving them vulnerable to institutional neglect, and easy prey for under-the-radar cost-savings.

Figures for total spending on mental health services vary depending on source and definition, inevitably leading to confusion, claim and counterclaim; although clearly of some importance, what matters more to core services is the detail. Nevertheless, analysis by the Royal College of Psychiatrists^7^ suggests that, despite claims that spending ‘is higher than ever’, total income (in today's prices) received by English mental health trusts in 2016–2017 was 1% less than in 2011–2012 (Scotland, Wales and Northern Ireland were 6, 0.3 and 1.3% less, respectively). An analysis by the British Medical Association^8^ concluded that despite geographical variations, there appears to be no obvious uplift in spending in recent years, noting concerns that government commitments to increased funding are not reaching front-line services, and a significant number of English Clinical Commissioning Groups are not meeting the Mental Health Investment Standard set out by NHS England. With the size of the overall cake barely changing, but several new and specialist (‘non-core’) services significantly expanding with ‘new’ investment, it seems likely that actual funding available to core services has decreased. Regarding the detail, what numerical and narrative features can be discerned within the core landscape, e.g. from NHS Benchmarking and the various other reports?

Between 2012 and 2017 there was a 17% reduction in adult acute beds, a 36% reduction in older adult beds and a 10% decline in acute adult admission rates.^9^ Average length of stay decreased slightly in adults and increased slightly in older adults. Adult bed occupancy (excluding leave) rose from 91 to 95% and involuntary admissions rose from 25 to 35%. In 2016–2017, delayed transfers of care increased to 5.4% of occupied bed days, up from 3.7% the year before. An analysis by the British Medical Association found that during 2016–2017, there were 5876 acute adult out-of-area placements for mental health treatment, a rise of 39% on 2014–2015.^10^ In May 2018, around 600 acute adult out-of-area placements, costing £9 million per month, were needed each month (in England) because of local bed unavailability.^11^

FYFVMH did include crisis teams aspirations: by 2020–2021, NHS England ‘should ensure that a 24/7 community-based mental health crisis response is available in all areas across England and that services are adequately resourced to offer intensive home treatment as an alternative to acute inpatient admission’.^1^ The latest monitoring (2016–2017) shows that only 23% of crisis resolution team services were able to meet selected core functions.^12^

The NHS nursing workforce has grown between 2010 and 2017, but the number of mental health nurses has declined by 12%,^13^ mostly within acute in-patient care as bed numbers have reduced, whereas the number of (mental health) community nurses has increased slightly. In generic CMHTs, overall staffing levels per population, perhaps surprisingly, seem to have been relatively stable in 2014–2017, although with some changes to the skill-mix. Before this, however, were at least 6 years of austerity, during which an unknown quantity of ‘easy, low-hanging fruit’ workforce cost-savings were likely made. Community case-load figures per population have declined slightly in the past 2 years: by 10% in older age CMHTs and 5% in working age CMHTs. Current data shows that for every 100 patients in community teams, there are only two qualified community psychiatric nurses. Generic CMHT referrals peaked in 2015–2016, but waiting times vary considerably and seem to be growing. More data (due October 2018) is needed to clarify trends, but the general sense is of CMHTs struggling to hold back the rising tide of demand in all its forms.

There are further complexities: many assertive outreach teams were reabsorbed into generic CMHTs (only 25% of trusts still reported assertive outreach teams/data in 2017), increasing CMHT clinical case-load intensity; how many of the staff also transferred is simply unclear. Vacancy rates are not benchmarked at all, whereas generic CMHT ‘cost improvement savings achieved’ are, remaining steady at 2–6% for most trusts. These were likely achieved, at least in part, by holding vacancies, which the King's Fund reports as currently about 10% across all mental health services;^13^ it also notes, ‘trusts must deliver annual cost-savings, and a key area for achieving this is workforce management’. Generic CMHTs, without any national policy priorities, no targets to measure and no ‘safer staffing’ safeguards, will remain highly vulnerable targets for ongoing cost-savings.

Looking ahead, the latest Health Education England mental health workforce planning for FYFVMH^14^ estimates that an additional 20 900 posts will be needed nationally (across all professions: qualified, support and admin, including 700 medical staff). However, the only core service mentioned is crisis teams (with no increase in medical staff); there is no mention at all of in-patient or CMHT services.

Capacity, however, is a complex concept, depending not just on workforce, but on a multitude of inter-related factors, including demand, productivity and influences from other systems. The raw numbers rarely tell the whole story; narrative is needed, and the clear message from the range of commentators is of ever-increasing pressures within core services that were already operating on, or at the margins of, full capacity. The Care Quality Commission notes ‘an unprecedented set of challenges – high demand, workforce shortages, unsuitable buildings and poor clinical information systems’.^6^ Other sources include 2017 NHS Mental Health Benchmarking, available via www.nhsbenchmarking.nhs.uk, which reports, rather ominously, that:

‘In recent years, concerns have been raised that the levels of community care have not risen as quickly as may be required to match the reduction in acute inpatient beds, and that provision may still not be at the levels needed…whilst safe staffing level requirements have benefitted the inpatient environment, unfortunately they have not helped staffing in the community whose responsibilities have increased in terms of caseloads and having to care for more unwell patients in community.’

With that background, caveats and all, what would front-line clinicians in core adult mental health services like the policy makers to hear, and do?

## Front-line messages

One answer comes forcefully from a 2017 Royal College of Psychiatrists survey of front-line clinicians, regarding perceived progress on the Acute Adult Psychiatric Care Commission recommendations (personal communication). It describes an ‘overwhelming consensus that the provision and quality of care is declining’, highlighting deep and widespread concerns over core services, including in-patient care, crisis teams and particularly CMHTs. There is a clearly perceived chronic and growing lack of service capacity, when matched to increasing demands (clinical, operational and regulatory).

The issues are familiar by now: raised clinical thresholds to enter services, and increasing severity and complexity within them; growing waiting times for CMHT care coordination; bed shortages and increasing use of the Mental Health Act 1983 (possibly linked to health inequalities for Black and ethnic minority groups^15^); increased acute out-of-area placements (https://www.bma.org.uk/news/2016/october/plan-to-reduce-discharge-distances); an increasingly stressed workforce and flagging morale.^16^ System changes compound the pressures, such as shrinking social care services and expanding roles like safeguarding. Attempted mitigation measures usually have a modest effect at best, whereas their unintended consequences can sometimes make things worse. Creative service redesigns have mostly confirmed that whatever the model, capacity (and probably continuity^17^) trumps configuration. Quality and productivity improvement activities, although clearly valuable, usually produce more gradual, longer-term gains, but too slowly to turn the current tide.

The FYFVMH simply did not take a whole-systems view of mental health services. Instead, it focused heavily on specialist areas such as liaison psychiatry, perinatal mental health, early intervention in psychosis services, child and adolescent mental health services, forensics and primary care psychological therapy. To their credit, these typically generated significant political attention through a clear and up-to-date evidence base (especially health economics research) or public attention via the media. In contrast, core severe mental health services seemed less newsworthy, less politically appealing and had a more limited, historical evidence research base (itself symbolic and symptomatic of long-term relative neglect). Yet it is precisely these core services where the vast majority of care for severe mental illness is delivered, forming the backbone and foundation of the whole service; if they are struggling, and overwhelming evidence and opinion suggests that they are, then the whole system will inevitably struggle too because sufficient capacity is needed in every part of the system.

Currently, the FYFVMH's blind-spot over core services risks a lack of Parity of Esteem within mental health going undetected under the policy radar. Furthermore, early progress in many of the more specialist areas may become undermined because core and specialist services are inevitably linked and interdependent. Child and adolescent mental health services patients grow up, with many still needing care; acute hospital liaison patients may be followed up in CMHTs; early intervention in psychosis often becomes ongoing intervention within a generic CMHT; perinatal care does not stay perinatal forever and acute mental health in-patient units and forensic services transfer patients both ways. Each recipient of specialist services should later be able to swiftly access quality care within a core generalist service, when needed, as should those who are referred straight from primary care, yet this is becoming more and more challenging, given the growing core pressures.

Finally, it is worth reflecting on why the core services, particularly CMHTs, currently feel so neglected. Historically (1990s), they were once the new expanding services, following the shift from asylums to community care, and they were considered progressive and attractive to work in. Around 2000, new investment was linked to new services (National Service Framework^18^ teams: crisis teams, assertive outreach and early intervention in psychosis), making these now the exciting teams to work in, with attention, enthusiasm and talent shifting away from the core CMHTs and in-patient wards.

Following the onset of austerity after 2008, alarm bells soon rang for the already drifting core, with senior clinicians noting that ‘demographic trends ensure that demand will rise and harsh economic realities dictate that resources will in real terms shrink’.^19^ Cost improvement plans inevitably followed, and rationalisation took place, with many assertive outreach teams merging back with CMHTs.^20^ The search got underway for any new service configurations that might be inherently more efficient; they were not especially (as noted before^21^), at least not in the prevailing climate.

Finally came the paradoxical pairing of ongoing austerity and an emerging Parity of Esteem agenda, both within a confusing commissioning environment, hampered by immature information systems. Priority areas grew, with commissioners and senior managers more focused on newer, ‘shiny’ services, whereas the older core, typified by CMHTs and in-patient units, were quietly considered ‘fair game’ for ongoing cost-savings (typically 3–6% each year), systematically slicing them to part fund the newer services. This gradual shift of resources away from core areas went largely unreported, hidden by limited informatics, minimal relevant national core targets, a chronic accommodation to the growing clinical risks and a lack of media appeal. Much like their patients, most core services, and CMHTs especially, have quietly remained out of sight and out of mind, a no-show in the FYFVMH calls for evidence, and therefore not making the policy cut. Front-line core staff were optimistically exhorted, ‘We've always made annual cost-savings before, so we know we can do it again’. But as with most simplistic rules of thumb, it only works for so long, and up to a point. Like anorexia, there comes a time when further safe reduction is simply not possible. For many core services, that point was probably reached some time ago.

## What to do?

Policy makers now need to publicly recognise the burgeoning crisis in core services. Any FYFVMH sequel must refocus policy more explicitly on CMHTs, crisis teams and in-patient care, rescuing, resuscitating and relaunching them all, along with improved information systems to support and monitor their regeneration.

There are glimmers of hope: the National Collaborating Centre for Mental Health ‘Mental Health Care Pathway: Community Mental Health Services’ project,^22^ nearing completion, arose from an FYFVMH recommendation to ‘establish comprehensive pathways and quality standards for the rest of the mental health system’. But it came with worrying limitations: they were last in line for development, waiting times were to be informed by clinical evidence (not targets), and they can be implemented as funding becomes available. Urgently addressing this wooliness would be a welcome start.

The potential benefits of reinvestment (and the risks of not reinvesting) are not limited to the core services, but extend to the whole wider system, even beyond mental health into general society.^23^

For too long now core services have been allowed to struggle along in a relative policy, priority and informatics vacuum, through which only muffled cries have so far travelled. Please, finally and quickly, will someone see the signs, hear their voice and begin to restore the foundations?

## About the author

**Dr Andrew Moore** is a consultant psychiatrist with North Devon Community Mental Health Services (Sector A Team), Devon Partnership NHS Trust, UK.

## References

[1] Independent Mental Health Taskforce. *The Five Year Forward View for Mental Health* NHS England, 2016 (https://www.england.nhs.uk/wp-content/uploads/2016/02/Mental-Health-Taskforce-FYFV-final.pdf).

[2] NHS England. *Mental Health Five Year Forward View Dashboard* NHS England, 2018 (https://www.england.nhs.uk/mental-health/taskforce/imp/mh-dashboard/).

[3] Public Health England. *Mental Health, Dementia and Neurology.* UK Government, 2018 (https://fingertips.phe.org.uk/profile-group/mental-health).

[4] Commission on Acute Adult Psychiatric Care. *Old Problems. New Solutions: Improving Acute Psychiatric Care for Adults in England.* Royal College of Psychiatrists, 2016 (https://www.rcpsych.ac.uk/pdf/Old_Problems_New_Solutions_CAAPC_Report_England.pdf).

[5] NHS Improvement. *Lord Carter's Review into Unwarranted Variations in Mental Health and Community Health Services* NHS England, 2018 (https://improvement.nhs.uk/about-us/corporate-publications/publications/lord-carters-review-unwarranted-variations-mental-health-and-community-health-services/).

[6] Care Quality Commission. *The State of Care in Mental Health Services, 2014 to 2017* Care Quality Commission, 2017 (https://www.cqc.org.uk/publications/major-report/state-care-mental-health-services-2014-2017).

[7] Royal College of Psychiatrists. *Mental Health Trusts’ Income Lower Than in 2011-12* Royal College of Psychiatrists, 2018 (https://www.rcpsych.ac.uk/mediacentre/pressreleases2018/mentalhealthtrustincome.aspx).

[8] British Medical Association (BMA). *Lost in Transit? Funding for Mental Health Services in England.* BMA, 2018 (https://www.bma.org.uk/collective-voice/policy-and-research/public-and-population-health/mental-health/funding-mental-health-services).

[9] NHS Benchmarking Network. *Mental Health Benchmarking.* NHS Benchmarking Network, 2016 (https://shsc.nhs.uk/wp-content/uploads/2017/02/Item-4iii-Benchmarking-of-MH-Services.pdf).

[10] British Medical Association (BMA). *BMA Figures Show Startling Rise in Mental Health Out of Area Placements.* BMA, 2018 (https://www.bma.org.uk/news/media-centre/press-releases/2017/june/bma-figures-show-starling-rise-in-mental-health-out-of-area-placements).

[11] NHS Digital. *Out of Area Placements in Mental Health Services.* NHS Digital, 2018 (https://files.digital.nhs.uk/A4/44D85A/oaps-rep-may-2018.pdf).

[12] NHS England. *Mental Health Five Year Forward View Dashboard.* NHS England, 2018 (https://www.england.nhs.uk/wp-content/uploads/2018/03/mhfyfv-dashboard-q1-q2-1718.xlsm).

[13] Gilburt H. *Funding and Staffing of NHS Mental Health Providers: Still Waiting for Parity* The King's Fund, 2018 (https://www.kingsfund.org.uk/publications/funding-staffing-mental-health-providers).

[14] Health Education England. *Stepping Forward to 2020/21: The Mental Health Workforce Plan for England* NHS England, 2017 (https://www.hee.nhs.uk/sites/default/files/documents/Stepping%20forward%20to%20202021%20-%20The%20mental%20health%20workforce%20plan%20for%20england.pdf).

[15] Monitoring the Mental Health Act in 2016/17, Care Quality Commission. (https://www.cqc.org.uk/publications/major-report/monitoring-mental-health-act-report).

[16] UNISON. *Struggling to Cope. Mental Health Staff and Services Under Pressure.* UNISON, 2017 (https://www.unison.org.uk/content/uploads/2017/10/Struggling-to-Cope.pdf).

[17] Pereira Gray DJ, Sidaway-Lee K, White E, Thorne A, Evans PH. Continuity of care with doctors—a matter of life and death? A systematic review of continuity of care and mortality. BMJ Open 2018; 8: e021161. 10.1136/bmjopen-2017-021161 PMC6042583 29959146

[18] Department of Health and Social Care. *National Service Framework: Mental Health* Department of Health and Social Care, 1999 (https://www.gov.uk/government/publications/quality-standards-for-mental-health-services).

[19] Holloway F. Gentlemen, we have no money therefore we must think’ – mental health services in hard times. Psychiatrist 2011; 35(3): 81–3.

[20] Rosen A, Killaspy H, Harvey C. Specialisation and marginalisation: how the assertive community treatment debate affects individuals with complex mental health needs. Psychiatrist 2013; 37: 345–8.

[21] Thornicroft G, Becker T, Holloway F, Johnson S, Leese M, McCrone P, Community mental health teams: evidence or belief? Br J Psychiatry 1999; 175: 508–13. 10789346 10.1192/bjp.175.6.508

[22] Royal College of Psychiatrists. *Mental Health Care Pathways.* Royal College of Psychiatrists, 2018 (https://www.rcpsych.ac.uk/workinpsychiatry/nccmh/mentalhealthcarepathways.aspx).

[23] Layard R. *A New Priority for Mental Health.* Centre for Economic Performance, London School of Economics and Political Science, 2015.

